# Highly Ductile and Ultra-Thick P-Doped FeSiB Amorphous Alloys with Excellent Soft Magnetic Properties

**DOI:** 10.3390/ma11071148

**Published:** 2018-07-06

**Authors:** Zongzhen Li, Shaoxiong Zhou, Guangqiang Zhang, Wei Zheng

**Affiliations:** 1 Advanced Energy & Materials Research Institute Co., Ltd., Changzhou 213000, China; zongzhenli@hotmail.com (Z.L.); zhangguangqiang@atmcn.com (G.Z.); 2 Central Iron & Steel Research Institute, Advanced Technology & Materials Co., Ltd., Beijing 100081, China; 3 Institute for Advanced Materials and Technology, University of Science and Technology Beijing, Beijing 100083, China; zhengwei@atmcn.com

**Keywords:** amorphous alloys, microstructure, soft magnetic properties, bending ductility

## Abstract

Herein, we demonstrate the successful synthesis of novel Fe_80_Si_9_B_(11−*x*)_P*_x_* (*x* = 0, 1, 3, 5, 7) ultra-thick amorphous ribbons by planar flow casting. The influence of P alloying on glass forming ability (GFA), microstructure, thermal stability, soft magnetic properties, and ductility has been systematically investigated. The results reveal that introduction of P into Fe_80_Si_9_B_11_ alloy can remarkably enhance the GFA and increase critical thickness (*t*_c_) of the alloy from 45 to 89 um. Furthermore, the annealed FeSiBP amorphous alloys exhibited excellent soft magnetic properties, including high saturation magnetic flux density of 1.54 T, the low coercivity of 1.5 A/m, and low core losses of 0.15 W/kg. In addition, the representative Fe_80_Si_9_B_8_P_3_ ultra-thick amorphous alloy demonstrate excellent ductility even after annealing at 400 °C for 10 min, which indicates the superior performance of P-doped FeSiB alloys as compared to the commercial Fe_78_Si_9_B_1__3_ (Metglas 2605 S2) alloy. The combination of high GFA, excellent ductility, and low core losses of newly developed FeSiBP amorphous soft magnetic alloys makes them attractive candidates for magnetic applications in the high-frequency and high-speed electric devices.

## 1. Introduction

With the development of science and technology, much effort has been devoted to making devices faster, lighter, and more energy efficient, which would make life easier or be more environmentally friendly [1,2,3,4,5]. The energy efficiency of various power electronic devices, such as transformers [5], electric vehicle [6], high-frequency, and advanced high-speed motors [7], could be significantly improved by using newly developed amorphous magnetic cores. The amorphous soft magnetic alloys do not possess crystal-like periodicity and symmetry and simultaneously exhibit superior mechanical characteristics, high corrosion resistance, and excellent soft magnetic properties [8]. Up to now, novel soft magnetic materials have been applied in many fields, such as energy storage, energy conversion, filtering, power generation, and sensing. It has been demonstrated that the replacement of conventional magnetic cores by amorphous soft magnetic cores in distribution transformers, used in electricity networks, can reduce no-load losses by more than 80% [9,10].

Despite the fact that silicon steel is the most commonly used soft magnetic material for motors, high power conversion, and distribution transformers, it exhibits a large amount of core losses at high-frequency for advanced high-speed devices. It has been reported that core losses results in substantial temperature rise and deteriorates the device performance [11]. Regarding core losses, amorphous materials possess higher electrical resistivity, when compared with silicon steels, and much less ribbon thickness of about 20 μm fabricating by using planar flow casting [12,13]. Hence, amorphous soft magnetic materials demonstrate lower core losses over a wide range of frequency. With the increasing demand for high-efficiency devices, the amorphous soft magnetic alloys have garnered significant research attention and have shown the ability to effectively reduce energy conversion loss. Generally, the wound amorphous soft magnetic cores, which are used in distribution transformers, have a flat geometry with a larger diameter and smaller axial length, which are easy to manufacture and require less cutting [14,15]. However, wound cores imply relatively high core losses and reduction of these core losses require further cutting. Moreover, wound cores are not suited for further applications as complex shaped magnetic components based upon these novel materials [16,17]. The geometrical optimization by cutting significantly enhances the power density when compared to the equivalent silicon steel components. However, the brittleness and relatively small thickness of conventional Fe-based amorphous soft magnetic alloys limit the potential applications and raise the technical challenges in manufacturing processes [13,18,19]. Therefore, the amorphous soft magnetic alloys with high thickness and better ductility should be developed for the successful realization of low-loss electrical devices.

The firstly commercial Fe_78_Si_9_B_1__3_ (Metglas 2605 S2) amorphous alloy is widely used in distribution transformers due to its excellent combination of low raw material cost and excellent soft magnetic properties, such as low coercivity, high saturation magnetization, and low core losses. Fe_80_Si_9_B_1__1_ (Metglas 2605 SA1) amorphous alloy was further developed subsequently, and the saturation magnetization was increased from 1.42 to 1.56 T [5,20]. However, the relatively poor glass forming ability (GFA) and fragility, after annealing, limits their potential applications. It has been reported that minor alloying is an effective technique to improve the GFA of Fe-based amorphous alloys [21,22,23,24]. More recently, Chang et al. have reported that incorporation of phosphorous (P) into Fe–Si–B alloys effectively enhanced the GFA of Fe_76_Si_9_B_10_P_5_ alloy, which resulted in the casting of the Fe_76_Si_9_B_10_P_5_ glassy rod with a critical diameter of 2.5 mm by conventional copper mold casting [25]. The excellent GFA of this newly developed Fe-based amorphous alloy opens up avenues for further research for the development of thick amorphous soft magnetic ribbons. As a matter of fact, the development of Fe-based amorphous alloys with excellent ductility, high strength, enhanced GFA, and desirable soft magnetic properties is challenging and vitally important for a wide range of applications. Moreover, after some pioneer studies, a large number of Fe-based bulk amorphous alloys have been synthesized mainly by copper mold casting [26,27,28]. Unfortunately, the reports on Fe-based ultra-thick amorphous ribbons are rare and the influence of minor-element alloying on ductility, core losses, and soft magnetic properties has not been explored in detail yet. The thermal stability of amorphous alloys must be considered for practical applications because of their tendency to transform from metastable states into more stable states when they are supplied with the necessary thermal energy [29,30,31]. Annealing is a common way of introducing this transformation of amorphous alloys, and their soft magnetic properties could be greatly improved by suitable annealing [32,33,34]. Herein, we have demonstrated the effects of P alloying and annealing on Fe_80_Si_9_B_(11−*x*)_P*_x_* (*x* = 0, 1, 3, 5, 7) alloys and systematically evaluated the GFA, thermal stability, soft magnetic properties, and ductility of newly developed FeSiBP ultra-thick amorphous ribbons.

## 2. Materials and Methods

### 2.1. Materials and Sample Preparation

**Figure 1:**
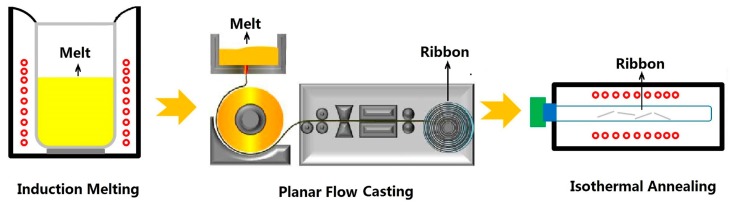
The schematic formation process for the Fe_80_Si_9_B_(11−*x*)_P*_x_* (*x* = 0, 1, 3, 5, 7) amorphous soft magnetic alloys.

The high-purity metals, such as Fe (99.99 wt.%), Si (99.99 wt.%), B (99.99 wt.%), and pre-alloyed Fe_3_P (99.9 wt.%) were used in this study. The schematic formation process for the Fe_80_Si_9_B_(11−*x*)_P*_x_* (*x* = 0, 1, 3, 5, 7) amorphous soft magnetic alloys is illustrated in Figure 1. Alloy ingots, with the nominal composition (in at.%) of Fe_80_Si_9_B_(11−*x*)_P*_x_* (*x* = 0, 1, 3, 5, and 7), were firstly prepared by the induction melting of a mixture of the high-purity metals in a Zr-gettered high-purity argon atmosphere. Subsequently, continuous Fe_80_Si_9_B_(11−*x*)_P*_x_* (*x* = 0, 1, 3, 5, 7) amorphous ribbons, with a width of 5 mm and a thickness of 25–90 μm, were produced through planar flow casting under atmospheric conditions. Finally, the ribbons were cut into 8 mm long strips, which were further annealed in a vacuum furnace and subsequently quenched in water.

**Figure 2:**
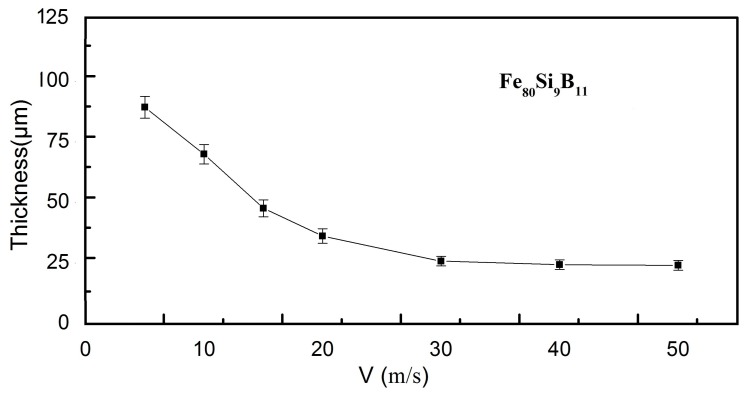
Variation of the thickness for Fe_80_Si_9_B_11_ as-spun ribbons as a function of the tangential wheel speed.

Each run was carried out by melting 15 g of the alloy ingots in quartz tubes having rectangular slit of 5 mm × 0.4 mm during the sample preparation processes. The castings were performed with tangential wheel speed from 5 to 50 m/s keeping the other production parameters constant: pressure injection, melt temperature, and the distance between nuzzle and wheel were kept at 400 kPa, 1300 °C, and 2 mm, respectively. The thickness of the melt-spin amorphous ribbons was controlled by changing the tangential wheel speed. The ribbon thickness decreased with increasing wheel speed from ~89 μm, corresponding to 5 m/s down to ~25 μm at 50 m/s for the representative Fe_80_Si_9_B_11_ as-spun ribbons, as illustrated in Figure 2. The ribbon thickness was measured by using an eddy-current coating thickness measurement gauge (HCC-25A).

### 2.2. Characterization Methods

The crystal structure and morphology of the as-spun ribbons were analyzed by using X-ray diffraction (XRD, Bruker D8 Advance, Billerica, MA, USA), with Cu-Kα radiations and high-resolution transition electron microscopy (HRTEM, TECNAI F20, FEI Company, Hillsboro, OR, USA), respectively. The saturation magnetization (*B_s_*) was measured by using a vibrating sample magnetometer (VSM, Lake Shore 7410, Carson, CA, USA) at a maximum applied field of 800 kA/m. The coercivity (*H_c_*) measurements were carried out by using a DC B-H loop tracer (RIKEN BHS-40, Tokyo, Japan) in a maximum applied field of 800 A/m. The core losses were measured by using an AC B-H loop tracer (Riken, Tokyo, Japan), which was in the frequency range of 50 to 1000 Hz, under the induction of 0.5 to 1 T. The mechanical characterization of the ribbons was carried out by bending the ribbons over the 5-mm mandrels. Bending tests were conducted on the ribbons by bending the ribbons around the mandrels with following diameters, listed in descending order, 20, 15, 10, 5, and 2 mm, according to ASTM Standard E 796-94 [35]. After each bending test, the sample was examined in the SEM for the characterization of shear bands. If the air-side of the ribbon sample did not exhibit shear bands, the process was performed repeatedly on the same sample, using another mandrel with a slightly smaller diameter, until the ribbon sample fractured or permanent deformation via shear band was detected on the outer surface. The ability to resist permanent deformation was measured by using Vickers diamond indenter (AKASHI, AVK-A, Tokyo, Japan) with a load of 2 N and a holding time of 5 s. The shear deformation was measured on the air-side of the sample, and surface was examined by using field emission scanning electron microscope (FESEM, FEI-Nova Nano-SEM-450, Hillsboro, OR, USA). All of the measurements were performed at room temperature.

## 3. Results

### 3.1. Glass Forming Ability (GFA) Analysis

**Figure 3:**
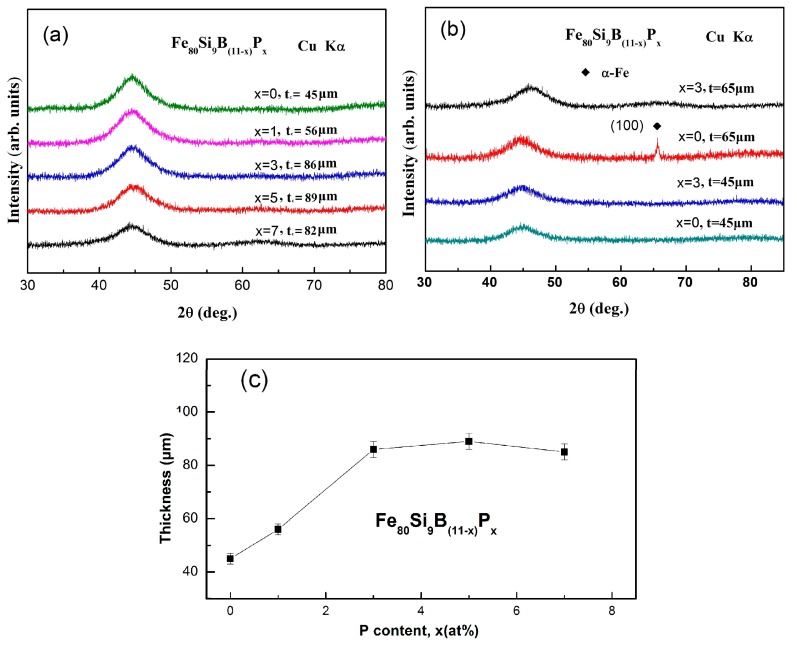
(**a**) X-ray diffraction (XRD) patterns of the as-spun Fe_80_Si_9_B_(11−*x*)_P*_x_* (*x* = 0, 1, 3, 5, 7) amorphous ribbons with critical thickness, (**b**) XRD patterns of melt-spun Fe_80_Si_9_B_(11−*x*)_P*_x_* (*x* = 0, 3) amorphous ribbons s with different thickness and (**c**) the variation of critical thickness (*t*_c_) as a function of P content for Fe_80_Si_9_B_(11−*x*)_P*_x_* (*x* = 0, 1, 3, 5, 7) melt-spun ribbons.

The effect of P substitution for B on the amorphous forming ability of the Fe_80_Si_9_B_(11−*x*)_P*_x_* (*x* = 0, 1, 3, 5, 7) ultra-thick amorphous ribbons has been determined by using critical thickness (*t*_c_). Figure 3a presents the XRD patterns and corresponding critical thickness of as-spun Fe_80_Si_9_B_(11−*x*)_P*_x_* alloy ribbons. The XRD measurements were carried out on air-side of the ribbons, where the cooling rate is the lowest. The XRD patterns exhibit broad diffraction spectra without distinct diffraction peaks, which indicates the amorphous nature of as-spun Fe_80_Si_9_B_(11−*x*)_P*_x_* alloy ribbons. The XRD patterns of the as-spun Fe_80_Si_9_B_(11−*x*)_P*_x_* (*x* = 0, 3) samples, with a thickness of 45 and 65 μm, are presented in Figure 3b for comparison. The broad halo peak demonstrates that both of the alloys exhibit amorphous phase when the thickness is 45 μm. However, when the thickness was increased to 65 μm, some tiny crystalline peaks corresponding to the α-Fe crystalline phase were observed for the alloys with *x* = 0, but the alloy with *x* = 3 still presents the fully amorphous structure. Glass forming ability (GFA) can be defined as the maximum amorphous thickness (for ribbons) or diameter (for rods) of a sample produced via quenching from the liquid state. In general, GFA exhibits an inverse relationship with the critical cooling rate and a direct relationship with the critical thickness of the sample [36]. The critical thickness (*t*_c_) as a function of P content for Fe_80_Si_9_B_(11−*x*)_P*_x_* (*x* = 0, 1, 3, 5, 7) melt-spun ribbons is showed in Figure 3c. The critical thickness (*t*_c_) of Fe_80_Si_9_B_(11−*x*)_P*_x_* alloy ribbons has shown a direct relationship with P content and found to be 45, 56, 86, 89, and 82 μm for *x* = 0, 1, 3, 5, and 7, respectively. Firstly, *t*_c_ slightly increased from 45 to 56 μm with an increase in P content from *x* = 0 to 1. *T*hen *t*_c_ increased sharply and reached a value larger than 80 μm for alloys with *x* = 3. Finally *t*_c_ kept a larger value high than 80µm with increasing *x* up to 7. The XRD results demonstrate that optimal P content can significantly enhance the GFA of the as-spun Fe_80_Si_9_B_11_ alloy ribbons.

**Figure 4:**
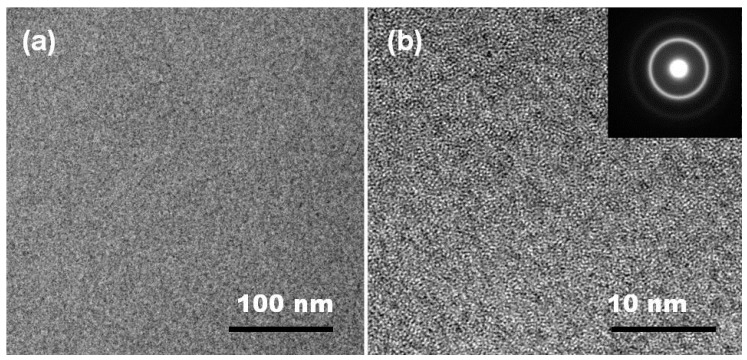
The Bright-filed TEM images (**a**) high-resolution TEM (HRTEM) images and (**b**) corresponding selective area diffraction patterns (inset) of Fe_80_Si_9_B_8_P_3_ sample with a ribbon thickness of 65 μm.

The microstructure of the as-spun Fe_80_Si_9_B_8_P_3_ sample with a ribbon thickness of 65 μm was further observed by TEM. The bright-field TEM image does not show any significant feature, as shown in Figure 4a, which is typical of an amorphous material. Moreover, the HRTEM image did not show the lattice fringes (Figure 4b), confirming the amorphous nature of the as-prepared sample. Furthermore, the corresponding SAED consists of a broad diffraction halo, along with a large faint halo, which further confirms the amorphous character of the as-spun Fe_80_Si_9_B_8_P_3_ sample. Hence, both the XRD and TEM results indicate that the GFA of Fe_80_Si_9_B_11_ alloy has been significantly improved with P doping (3 at.%).

**Figure 5:**
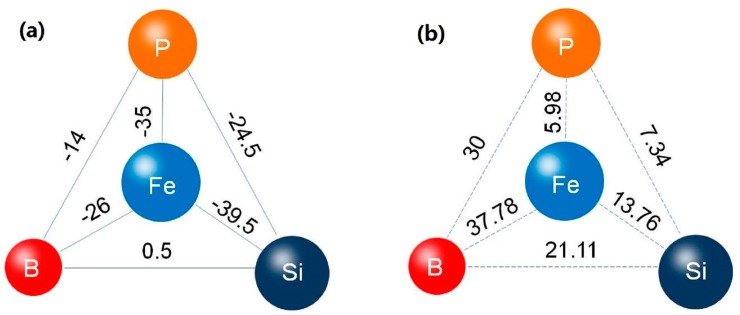
Schematic illustration for the mixing enthalpy (**a**) and mismatch in atomic radius (**b**) for the Fe-Si-B-P alloy system.

The GFA of an alloy is determined by the composition of the base alloy, purity of the constituents and nature of the alloying additions. It has been reported that atomic size difference and the thermodynamic properties determine the effectiveness of the alloying addition on GFA [24,37]. In the present alloy system, as illustrated in Figure 5a, the mixing enthalpy of Fe-P, Si-P, Fe-B, and Si-B is −31, 0, −11, and 18 kJ/mol, respectively. It is obvious that the introduction of P leads to a larger negative mixing enthalpy, which enhances the GFA of the propose alloy system. On the other hand, P alloying results in higher atomic size mismatches between constituent elements, as shown in Figure 5b, which is also beneficial for GFA of alloy system [36,38,39].

### 3.2. Crystallization Characteristics

**Figure 6:**
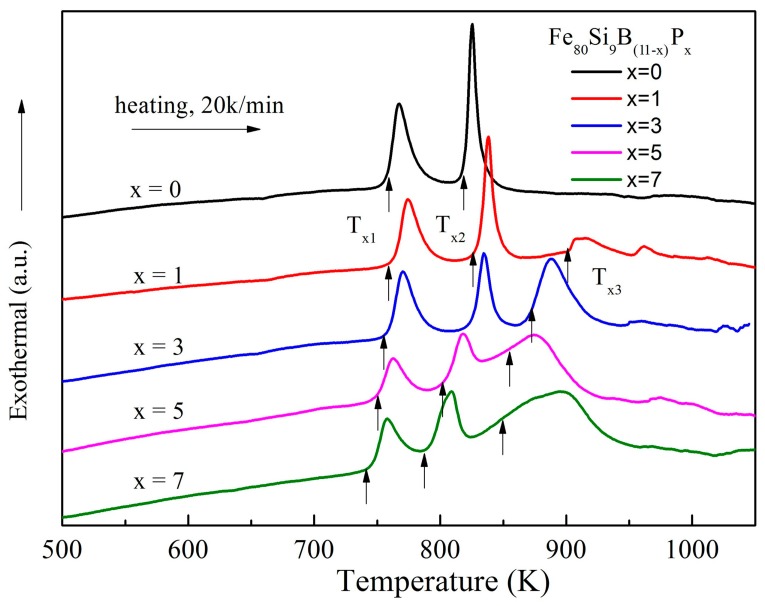
DSC curves of the as-spun Fe_80_Si_9_B_(11−*x*)_P*_x_* (*x* = 0, 1, 3, 5, 7) amorphous ribbons at a heating rate of 20 K/min.

The influence of P addition on the crystallization behavior of the as-spun Fe_80_Si_9_B_(11−*x*)_P*_x_* (*x* = 0, 1, 3, 5, 7) amorphous ribbons was also evaluated. DSC curves of all the ribbon samples at a heating rate of 20 K/min in Ar flow is shown in Figure 6. Each sample exhibited no distinct glass transition before crystallization. Two separate exothermic peaks were detected for P-free alloy, indicating that the crystallization occurred in two stages, and the corresponding peaks (marked as “P_1_” and “P_2_”) were located distantly. For the alloys with P alloying another exothermic peak P_3_ appeared, leading to the crystallization procedure taken place through three exothermic peaks. With P content increasing from 3 to 7 at.%, the two peaks got closer, which indicated the increase of thermal stability of the supercooled liquid. The deduction was verified by the fully amorphous characteristic in Figure 3 of the Fe_80_Si_9_B_8_P_3_ sample with a ribbon thickness of 65 μm. When compared with the well investigated Fe_80_Si_9_B_11_ alloy, which only could be made into ribbon sample with thickness less than 45 μm, it is strongly confirmed that 3 at.% P element addition can effectively improve the GFA of FeSiBP alloys.

### 3.3. Soft Magnetic Characteristics

**Figure 7:**
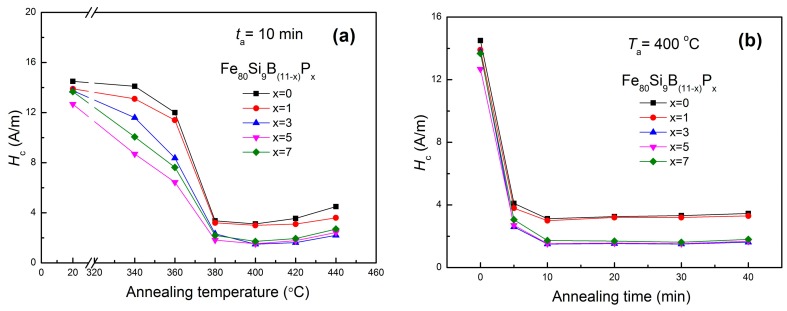
Annealing temperature (*T*_a_) dependence (**a**) and annealing time (*t*) dependence (**b**) of *H*_c_ for Fe_80_Si_9_B_(11−*x*)_P*_x_* (*x* = 0, 1, 3, 5, 7) amorphous alloys.

The planar flow casting (PFC) is one of the most promising and commonly used processes for the manufacture of amorphous ribbons. It is well known that the stresses are introduced into as-spun amorphous alloys during rapid casting [40,41]. Being one of the most important soft magnetic parameters coercivity (*H*_c_) is very sensitive to the residual stresses, which can be removed by structural relaxation during annealing. The variation of coercivity (*H*_c_) during annealing reflects the structural relaxation processes in amorphous phase [42,43,44]. Therefore, the dependence of *H*_c_ on annealing temperature (*T*_a_) and annealing time (*t*_a_) for as-spun Fe_80_Si_9_B_(11−*x*)_P*_x_* amorphous alloys is systematically studied. Figure 7a presents the relationship between annealing temperature and coercivity when the annealing time is fixed at 10 min. When *T*_a_ was increased from room temperature to 380 °C, the *H*_c_ gradually decreased due to stress releasing by the structural relaxation of the amorphous alloys. Then, *H*_c_ kept almost constant in the temperature range of 380–420 °C, because the amorphous alloys has transformed from metastable states into a more stable state and cast-in stresses during rapid casting has been fully released at this temperature range. However, the *H*_c_ value slightly increased up at 440 °C. Hence, an optimal temperature of 400 °C was selected for further experimentation, where minimum *H*_c_ value was observed. Figure 7b presents the dependence of *H*_c_ on annealing time (*t*_a_), measured at an optimal annealing temperature of 400 °C. The *H*_c_ values decreased with the increase of annealing time due to stress relaxation and attained a minimum value of 1.5 A/m after 10 min. Then, the *H*_c_ exhibited a little variation with respect to annealing time (*t*_a_) up to 40 min. Therefore, the optimal annealing temperature of 400 °C and an annealing time of 10 min were selected for further experiments.

**Figure 8:**
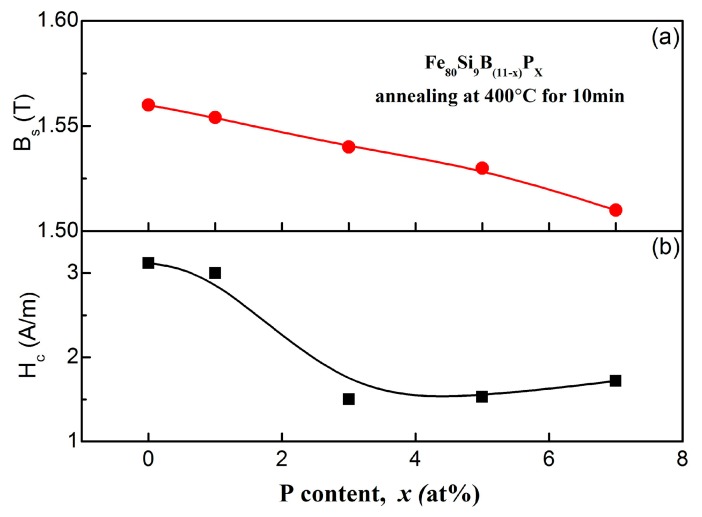
Changes of *H*_c_ (**a**) and *B*_s_ (**b**) for Fe_80_Si_9_B_(11−*x*)_P*_x_* (*x* = 0, 1, 3, 5, 7) amorphous ribbons as a function of P content annealed at 400 °C for 10 min.

The dependence of P content on *H*_c_ and *B*_s_ on Fe_80_Si_9_B_(11−*x*)_P*_x_* amorphous ribbons, after annealing under optimal conditions, is shown in Figure 8. First, the *H*_c_ values slowly decreased from 3.2 to 3 A/m, with an increase in P content from *x* = 0 to 1, respectively. Then, the *H*_c_ values drastically decreased and reached a minimum value of 1.5 A/m at *x* = 3 and it remained almost stable up to *x* = 7. On the other hand, the *B*_s_ values monotonically decreased with the increase of P content, which can be attributed to the reduced Fe content with higher P substitution. It should be noted that the minimum value of *H*_c_ (1.5 A/m) of the amorphous alloys is much less than the commercial Fe_80_Si_9_B_11_ (Metglas 2605 SA1) alloy. Low *H*_c_ favors the application of amorphous alloy core in high frequency and high-speed electrical devices. The enhanced GFA leads to the higher degree of amorphousness, a lesser number of domain wall pinning sites, which result in lower *H*_c_ values [45,46]. In summary, the soft magnetic properties of as-spun Fe_80_Si_9_B_(11−*x*)_P*_x_* amorphous ribbons can be optimized with optimal annealing conditions and P content.

**Figure 9:**
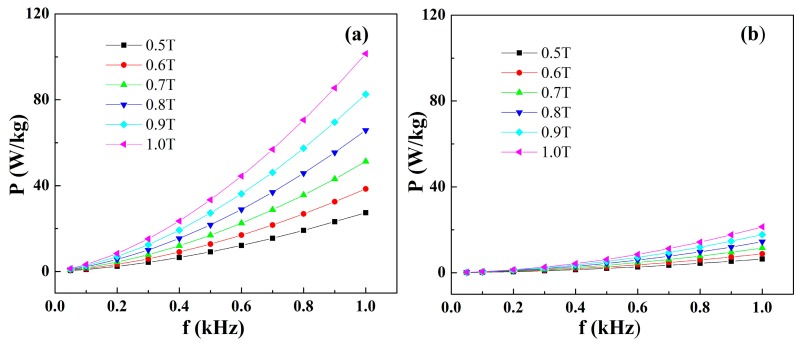
Core losses as a function of frequency in different induction; (**a**) conventional silicon steel (**b**) Fe_80_Si_9_B_8_P_3_ amorphous alloys.

The core losses will become heat dissipating and deteriorate the efficiency and stability of the magnetic devices. Thus, the core losses of these newly developed Fe-based amorphous alloys should be evaluated before practical applications. The core losses dependence on induction and frequency for conventional silicon steel and Fe_80_Si_9_B_8_P_3_ amorphous alloy is shown in Figure 9a,b. The core losses have shown a direct relationship with induction and frequency for both Fe_80_Si_9_B_8_P_3_ amorphous ribbons and silicon steel. However, the increasing rate of core losses in Fe_80_Si_9_B_8_P_3_ amorphous ribbons is much lower than silicon steel, particularly at high frequency. It is noteworthy that the Fe_80_Si_9_B_8_P_3_ amorphous alloy exhibited extremely low core losses values. The P1.0/50 (core loss at 50 H_Z_ in 1.0 T induction) for Fe_80_Si_9_B_8_P_3_ amorphous alloy is only 0.15 W/Kg. The dominant losses of magnetic cores are the hysteresis loss, which strongly depends on the *H*_c_. Hence, the Fe_80_Si_9_B_8_P_3_ amorphous alloy can be used in high induction (1.0 T) applications, due to extremely low *H*_c_, high *B*_s_ and low core losses. It is worthy to note that such extremely low core losses are uncommon for Fe-based amorphous alloys when compared with previous reports [8,20,25,47]. It has been required to prepare soft magnetic materials with lower core losses, combined with good soft magnetic properties, high-productivity, and low materials cost. Therefore, the present Fe–B–Si–P amorphous alloys are considered to be an important candidate for magnetic core materials.

### 3.4. Microstructural Analysis

**Figure 10:**
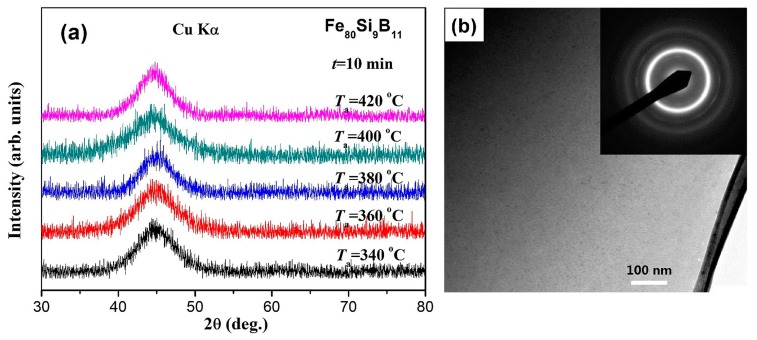
(**a**) X-ray diffraction (XRD) patterns of the as-spun Fe_80_Si_9_B_11_ samples annealed at different temperatures for 10 min; (**b**) The Bright-filed TEM images and corresponding selective area diffraction patterns (inset) of Fe_80_Si_9_B_11_ sample annealed at 420 °C for 10 min.

In order to further understand the effect of annealing on the variation of coercivity (*H*_c_), The characterized microstructure of the Fe_80_Si_9_B_11_ amorphous ribbons annealed at different temperatures for 10 min is shown in Figure 10. The XRD patterns taken from the air-side of the specimens annealed at 340–420 °C for 10 min are shown in Figure 10a. All of the XRD patterns reveal only a typical halo peak, and no peaks corresponding to crystalline phases, illustrating the amorphous nature of the ribbons after annealing. The Fe_80_Si_9_B_11_ ribbon annealed at 420 °C for 10 min was then subjected to TEM studied. Figure 10b shows its high-resolution TEM (HRTEM) image and the inset is the corresponding selected area electron diffraction (SAED) pattern. The HRTEM image shows a fully random arrangement of atoms, while the diffraction pattern consists of halos. These confirm that the annealed specimen is still fully amorphous. It is well known that glass formation ability (GFA) exhibits a direct relationship with the liquid thermal stability of amorphous alloys. Crystallization phase is more difficult to precipitate for the amorphous alloys with higher glass forming ability. When considering that the representative Fe_80_Si_9_B_11_ amorphous alloy with the lowest GFA still retains its amorphous nature even after annealing at 420 °C for 10 min, it can be safely concluded that all of the Fe_80_Si_9_B_(11−*x*)_P*_x_* samples could preserve amorphous microstructure during the annealing process at 340–420 °C for 10 min. Suitable annealing well below the crystallization point relaxes the structure, maintaining its amorphous character, and resulting in moderate magnetic softening due to the relief of intrinsic cast-in stresses during rapid casting.

### 3.5. Mechanical Characteristics

**Figure 11:**
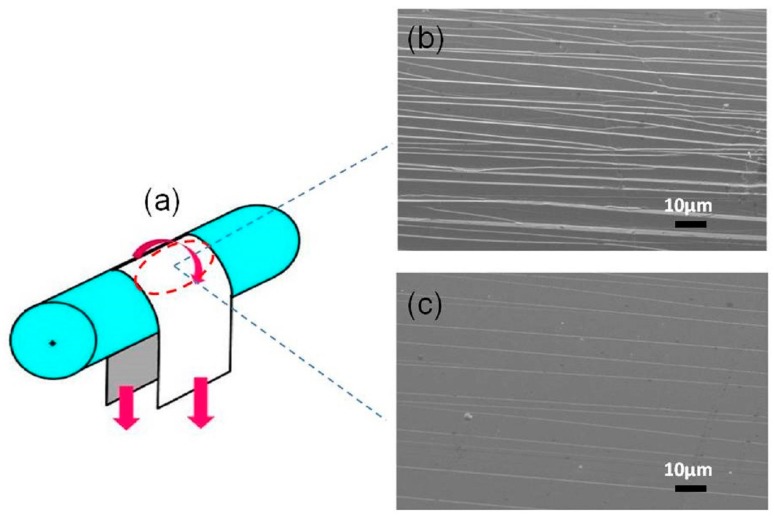
Schematic diagram of the bending test (**a**) and SEM images obtained from the top surface of the bent Fe_80_Si_9_B_8_P_3_ alloy ribbons; (**b**) as-quenched specimen; and, (**c**) annealed at 400 °C for 10 min.

The mechanical characterization, such as bending ductility and Vickers indentation tests, were carried out to explore the influence of P alloying and annealing conditions on mechanical properties of the representative Fe_80_Si_9_B_8_P_3_ amorphous ribbons. The Mandrel bend testing is illustrated in Figure 11a. Figure 11b,c exhibits the top surface SEM images of as-spun ribbons and samples annealed at 400 °C for 10 min after the bending test, respectively. As shown in Figure 11b, high-density shear bands and the corresponding interactions are visible on the specimen surface of the as-spun amorphous ribbons, implying the ductile nature of the specimen. It is consistent with the excellent bending ductility of as-spun amorphous ribbons, which can be bent to 180° without breaking. After annealing, the number of shear bands was reduced, whereas the inter-shear band spacing became larger, which indicates the reduction in ductility. However, we have not observed any cracks in the annealed Fe_80_Si_9_B_8_P_3_ specimen, which confirms the excellent bending ductility after annealing, as illustrated in Figure 11c.

**Figure 12:**
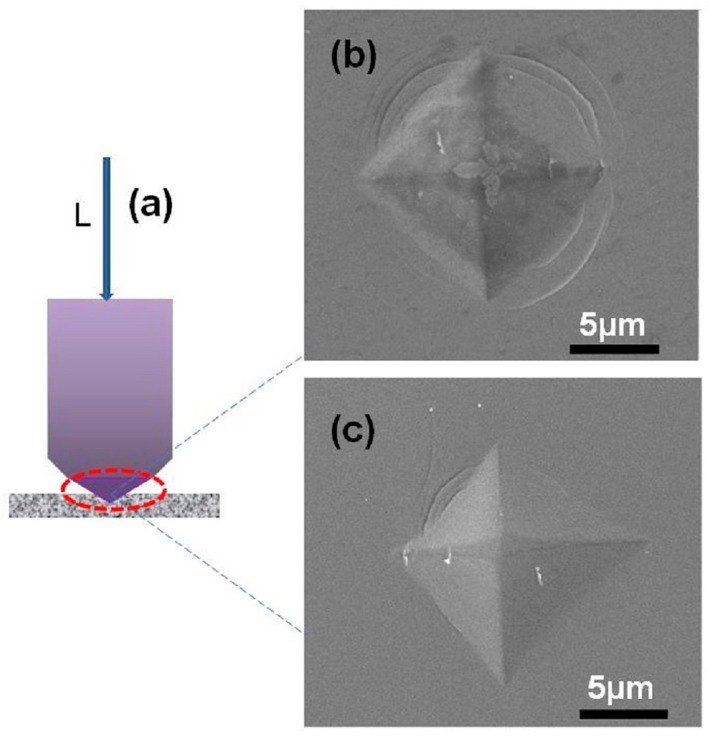
Schematic diagram of the Vickers indentation testing (**a**) and SEM images of indentation marks of the Fe_80_Si_9_B_8_P_3_ amorphous ribbon; (**b**) as-quenched specimen; and, (**c**) annealed at 400 °C for 10 min.

The indentation testing is illustrated in Figure 12a. The top-view of indentation marks of as-spun ribbons and samples annealed at 400 °C for 10 min, under the load of 2 N and a holding time of 5 s by Vickers indenter, are shown in Figure 12b,c. The Vickers hardness values of the as-cast and annealed Fe_80_Si_9_B_8_P_3_ amorphous ribbons are 970.4 and 950.8 HV, respectively, which are higher than that of the traditional silicon steels and exhibit the excellent wear resistance of prepared alloys [48]. The pile-up feature, near the indentation mark, suggests the inhomogeneous deformation of the samples. It was interesting to observe the numerous shear bands in the pile-up areas, around the hardness indentation marks, in both as-spun and annealed samples, which indicates the excellent deformation ability against indentation. However, the number and appearance of the shear bands, around the indentation marks, were lower and less obvious in annealed samples. The variation in the appearance of the indentation marks demonstrates that annealing reduced the ductility of Fe_80_Si_9_B_8_P_3_ amorphous ribbons. However, the presence of shallow slip-steps markings near the indentation and the absence of obvious cracks indicate that the samples still possess enough elastic-plastic deformation ability.

The atomic packing density is one of the most important factors affecting the ductility of amorphous alloys [49]. In Fe-Si-B-P alloy system, P was introduced as an alloying element and the dominant Fe element has a large atomic size mismatch of 6%, 38%, and 14% with Si, B, and P, respectively, as illustrated in Figure 5b. It has been reported that elements with larger atomic size mismatches can form a highly packed atomic configuration [50,51]. Hence, the incorporation of smaller size P element led to a much denser atomic packing arrangement, which promotes the slip and interaction of the shear bands and improves the ductility.

## 4. Conclusions

A comprehensive investigation was carried out to explore the influence of P alloying on glass forming ability (GFA), microstructure, soft magnetic properties, and ductility of Fe_80_Si_9_B_(11−*x*)_P*_x_* (*x* = 0, 1, 3, 5, 7) amorphous alloys. The main findings of this study are summarized, as follows:

1. The GFA and soft magnetic properties of as-spun Fe_80_Si_9_B_(11−*x*)_P*_x_* amorphous alloys were improved due to the incorporation of a minor amount of P element. However, the excess of P led to the reduction of iron content and the deterioration of saturation magnetization. Hence, an optimal amount of alloying element is required to attain desired properties.

2. The Fe_80_Si_9_B_(11−*x*)_P*_x_* amorphous alloys possess superior soft magnetic properties, such as high *B*_s_ of 1.54 T low *H*_c_ of 1.5 A/m and low core losses of 0.15 W/kg.

3. The representative Fe_80_Si_9_B_8_P_3_ amorphous alloy has exhibited excellent bending ductility and a ductile indentation response, even after annealing at 400 °C for 10 min, which demonstrates the good manufacturability of the as-spun and annealed Fe_80_Si_9_B_8_P_3_.

4. The combination of high GFA, low core losses, and excellent ductility, even after annealing, of FeSiBP ultra-thick amorphous ribbons, make them desirable for the industrial production and utilization as soft magnetic materials.
